# Chiral Plasmonic Biosensors

**DOI:** 10.3390/bios8040120

**Published:** 2018-12-01

**Authors:** Vladimir E. Bochenkov, Tatyana I. Shabatina

**Affiliations:** Chemistry Department of Lomonosov, Moscow State University, 119991 Moskva, Russia; tatyanashabatina@yandex.ru

**Keywords:** chirality, chiral plasmonics, LSPR, chiral biosensors

## Abstract

Biosensing requires fast, selective, and highly sensitive real-time detection of biomolecules using efficient simple-to-use techniques. Due to a unique capability to focus light at nanoscale, plasmonic nanostructures provide an excellent platform for label-free detection of molecular adsorption by sensing tiny changes in the local refractive index or by enhancing the light-induced processes in adjacent biomolecules. This review discusses the opportunities provided by surface plasmon resonance in probing the chirality of biomolecules as well as their conformations and orientations. Various types of chiral plasmonic nanostructures and the most recent developments in the field of chiral plasmonics related to biosensing are considered.

## 1. Introduction

Recent progress in plasmonics, the modern field of science dealing with resonant interaction of light with electrons at metal/dielectric interface, has strongly influenced disciplines such as physics, chemistry, and biotechnology by opening the new exciting possibilities for light manipulation at the nanoscale [1,2]. The unique property of surface plasmon resonance (SPR) to generate strongly amplified evanescent fields near metal surface has revealed new opportunities for label-free refractive index sensing [3], which has ultimately led to the commercialization and wide-spreading of the SPR detection technique, widely used for studying biomolecular interactions nowadays.

Plasmon-induced strong evanescent fields affect various optical processes in molecular systems and thus have found numerous applications in enhanced spectroscopic techniques, such as Surface-Enhanced Raman Scattering (SERS), Plasmon-Enhanced Fluorescence (PEF), Surface-Enhanced Infrared Absorption (SEIRA), etc. Various plasmonic nanostructures have been used as signal amplifiers and transducers for optical sensing. These plasmon-enhanced spectroscopic methods have been described in several comprehensive reviews [4,5,6].

A large fraction of naturally occurring biomolecules, including amino acids, sugars, and nucleotides, are chiral and exist only in one handedness. This stresses the paramount importance of stereoselective synthesis as well as of detecting and separating enantiomers, especially in pharmaceutics, since enantiomers of drug substances can have no therapeutic effect or can even be hazardous.

Molecular chirality can manifest itself by interaction with chiral electromagnetic fields as differential absorption of left- and right-handed circularly polarized light (CPL), referred as circular dichroism (CD); or by rotation of the polarization plane of the linearly polarized light, termed optical rotatory dispersion (ORD). These effects comprise the most common chiroptical responses and are related, one defining another through Kramers-Kronig relations [7]. Typically, chiroptical signals are very weak, so a large number of chiral molecules is needed for their experimental detection.

Due to the success of other plasmon-enhanced spectroscopic techniques, there is currently a high interest in chiral plasmonics, especially in using plasmon-enhanced near fields to increase the sensitivity of chiroptical spectroscopy [7,8,9]. Many chiral plasmonic nanostructures are being fabricated and studied both experimentally and theoretically. Several extensive reviews on the nature of plasmonic chirality and chiroptical effects in plasmonic nanostructures have been published recently [10,11,12].

This short review is devoted to chiral biosensing, leaving most of the fundamental aspects of plasmonic chirality as well as the fabrication details beyond its scope. In the following sections, we consider the main types of molecular–plasmonic systems capable of generating enhanced chiroptical signal, focusing on the recently published results of using plasmon-induced evanescent fields to detect the presence of certain biomolecules and (in some cases) to probe their orientation and high-order structure.

## 2. Molecular CD Enhanced by Exciton-Plasmon Coupling

Interaction of an achiral excitonic system with plasmonic particles can drastically enhance the absorbance signal due to the antenna effect [13]. Similar interactions can affect chiroptical response of a chiral molecular system coupled to a plasmon, ultimately leading to the stronger chiroptical response needed for biosensing.

### 2.1. Chiral Molecules Coupled to a Single Plasmonic Nanoantenna

One of the first experimental observations of strong (about two orders of magnitude) plasmonic CD enhancement has been reported for a system of colloidal silver nanoparticles with a chromophore molecule absorbing light at the same wavelength as the Localized Surface Plasmon Resonance (LSPR) of the nanoparticles [14].

**Figure 1:**
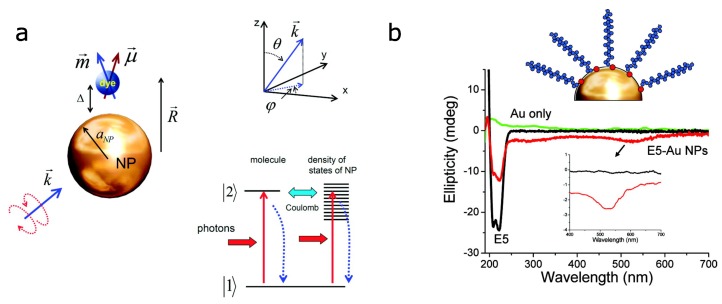
Circular dichroism of coupled molecular-plasmonic systems: (**a**) Schematics of the metal nanoparticle–dye molecule assembly used for theoretical calculations (left) and schematics of quantum transitions (right) with solid vertical (horizontal) arrows representing light (Coulomb)-induced transitions. The dotted vertical arrows are the relaxation processes. Reproduced with permission from Govorov et al. [15] Copyright 2010 American Chemical Society. (**b**) Illustration of gold nanoparticle surface covalently linked to the E5 peptide via the thiol linkage (red circles) and the CD spectra of E5 peptide (black line), gold nanoparticles (green line) and their assembly (red line). Reproduced with permission from Slocik et al. [19] Copyright 2011 American Chemical Society.

These results promoted theoretical studies of the nature of plasmonic CD enhancement [15,16,17,18]. It has been shown that coupling of a chiral molecule to a small achiral plasmonic nanoparticle (particle size ≈ 10 nm) can lead to the enhancement of the molecular CD signal combined with the appearance of a new CD signal at the wavelength of the plasmon resonance. The schematics of the studied model is presented in Figure 1a. Due to the small separation between the molecule and the nanoparticle, the Coulomb interaction between their electronic systems can be strong. As a result, the CD signal induced by molecular transition can be boosted by plasmon resonance, and, at the same time, the surface current in plasmonic nanoparticle becomes chiral due to the chiral molecule, resulting in a new CD signal [15].

The near-field enhancement decreases with the separation Δ between the metal surface and the molecule as $\Delta^{-3}$. The appearance of a new CD peak is important from the practical point of view, since most biomolecules have CD in the UV range and the appearance of the new CD signal in the visible range can be easily detected.

Several later experimental works proved these findings. For instance, an adsorption of peptide molecules on spherical gold nanoparticles with 10 nm radius induced a clear CD signal in the visible range of the spectrum [19]. Figure 1b schematically shows the system and the CD spectra. Similar results have been obtained for a bilayer of riboflavin 5${}^{\prime }$-monophosphate and polylysine molecules adsorbed on gold island films [20]. In this work, by careful control of the separation between the molecule and the metal nanoparticle, the authors were able to confirm the near-field enhancement of the induced CD signal. It has been noted that the adsorbed chiral molecules did not have to be in resonance with particles’ plasmon for this new CD peak to appear. However, matching the wavelengths of molecular absorption and plasmon resonance increased the effect, suggesting the use of nanostructures with plasmon resonance in the UV for biosensing [21,22]. The enhanced chiroptical response was observed for cystein and its derivatives in the presence of 45 nm spherical silver nanoparticles [23], for glutathione molecules adsorbed on 45 nm silver nanocubes [24] and for tobacco mosaic virus uniformly covered by spherical 5 nm gold nanoparticles [25].

It has been shown that the induced CD can be sensitive to the orientation of the transition dipole moments of the adsorbed molecule relative to the local plasmonic polarization of the nanoparticle [26]. The CD enhancement factors of two orders of magnitude was observed for gold/silver core-shell nanocubes of 42±2 nm covered by DNA molecules [21].

Thus, the use of a plasmonic antenna can be used to enhance the CD signal of small chiral molecules. However, the moderate CD enhancement and its strong sensitivity on the separation distance limits the broad application of this approach in biosensing.

### 2.2. Chiral Molecules in the Gap of a Plasmonic Dimer

**Figure 2:**
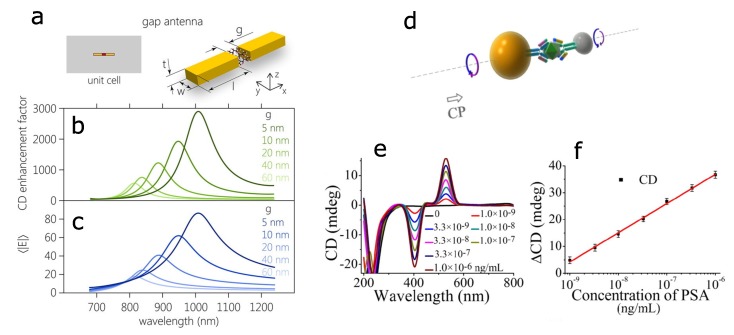
Chiral molecules in the gap of a plasmonic dimer: (**a**–**c**) theory and (**d**–**f**) experiment. (**a**) Schematics of gold nanorod dimer with chiral molecules immobilized in the gap; (**b**) calculated CD enhancement factor for different gap size; (**c**) corresponding values of average electric field in the gap volume. Reproduced with permission from Nesterov et al. [31] Copyright 2016 American Chemical Society. (**d**) Schematics of the Au–Ag heterodimer bridged by immunocomplex with prostate-specific antigen (PSA); (**e**) the CD spectra for different concentrations of PSA; (**f**) the CD calibration curve for PSA detection as a function of logarithmic PSA concentrations. Reproduced with permission from Wu et al. [32] Copyright 2013 American Chemical Society.

Stronger effect has been predicted for plasmonic “hotspots”, located within the gaps of dimer nanoantennas for both resonant and off-resonant CD signals [27,28,29,30]. The enhancement factors as high as 3000 could be expected for the gap size of 5 nm. The CD enhancement factor correlates with averaged electric field in the gap, as indicated by Figure 2a–c [31].

Experimentally, this effect was observed in several systems for aggregated metal nanoparticles, such as oligonucleotide-conjugated spherical gold nanoparticles [33,34], cysteine-modified gold nanorods [35,36] and nanospheres [37,38], as well as cholate-coated silver nanoparticles [39], etc. The CD signal has been strongly enhanced by the agglomeration of the particles, leading to the formation of nanometer-sized gaps.

A working demonstration of chiral biosensing based on the formation of asymmetric plasmonic dimers has been reported [40]. In this work, an antigen–antibody recognition reaction has been used to initiate self-assembly of gold nanorods, leading to the generation of detectable CD signal. The detection of bisphenol A with the detection limit of 0.02 ng/mL has been demonstrated.

The use of highly specific interactions between surface-immobilized complementary DNA primers is a very convenient way to assemble nanoplasmonic dimers with tailorable chiroptical response for application in biosensorics [34]. With some modifications, this approach has successfully been applied for the detection various analytes. For instance, silver ions could be detected with a limit of 2 pM by triggering the nanodimer assembly reaction [41]. The methyltransferase activity and inhibition has been studied using DNA-based chiroplasmonic assemblies of gold nanoparticles and endonuclease HpaII [42]. Constructed in a similar way, a heterogeneous system with Au core and Ag satellites has been used for the detection of ochratoxin A. The detection limit as low as 0.15 pg/mL has been reported [43]. Multimetal shell-engineered assemblies have been used to demonstrate the zeptamolar DNA detection [44].

Another sensing scheme has been presented recently for the determination of alpha-fetoprotein (AFP), which can serve as a marker of liver cancer [45]. The system has been constructed by AFP aptamer hybridization with its complementary sequence immobilized on gold nanoparticles, thus forming NP dimers, which display strong chiroptical activity. When AFP has been added, it binds strongly to the aptamer, resulting in destruction of the aptamer-DNA hybrid. The decrease in CD signal has been measured, and the low AFP detection limit of 11 pg mL${}^{-1}$ has been reported.

The same strategy has been applied to detection of 8-hydroxy-2${}^{\prime }$-deoxyguanosine, a well-known biomarker for oxidative DNA damage, in human serum [46]. Again, CD intensity showed log-linear correlation with concentration of the analyte molecules, and the detection limit of 33 pM.

Self-assembled gold heterodimers can be used to monitor intracellular concentration of analyte molecules. Thus, the detection of adenosine-5${}^{\prime }$-triphosphate in living cells with a limit of detection of 0.2 mM has been reported recently [47]. Telomerase activity has been studied and a limit of detection of $1.7\times 10^{-15}$ IU in a single HeLa cell has been determined [48].

Another general approach for constructing sensitive plasmonic dimers is by using antibody-antigen interactions, as depicted in Figure 2d–f. The chiroplasmonic detection of an environmental toxin, microcystin-LR, and a cancer biomarker, prostate-specific antigen (PSA), has been demonstrated using a silver–gold nanoparticle heterodimer [32]. The PSA detection limit of $5\times 10^{-10}$ ng/mL ($1.5\times 10^{-20}$ M) has been reported.

Recently, the detection of DNA molecules with concentrations below 100 pM has been successfully demonstrated by plasmon-induced CD from molecules located within the carefully arranged hotspot between two gold nanoparticles [49]. The influence of the particles shape, gap size etc., has been analyzed.

Thus, plasmonic dimers can be used for detection of small chiral molecules as well as enzymes and proteins. The high sensitivity and selectivity due to the use of DNA for biorecognition combined with the possibility of intracellular detection makes this approach highly promising for biomedical applications.

## 3. Chiral Assemblies of Achiral Plasmonic Nanoparticles

Another sensing approach is based on geometrical chirality of chiral molecules. Their assemblies with achiral plasmonic nanoparticles can generate chiroptical response not only due to exciton–plasmon interaction, as described in Section 2, but also by plasmon-plasmon coupling between nanoparticles, arranged in a chiral superstructure [50]. In the latter case, the molecules play a role of a template, defining handedness of the resulting assemblies. Thus, the presence of the molecules can be detected by the appearance of the CD signal.

**Figure 3:**
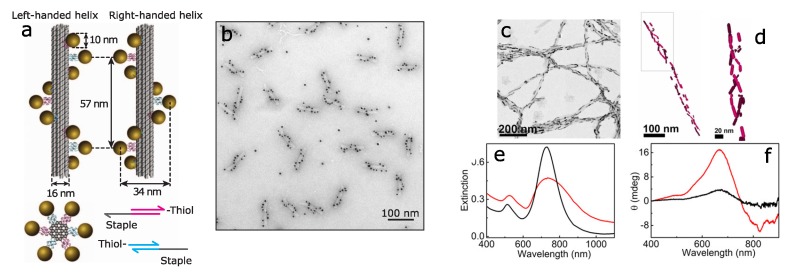
Biomolecule-assisted chiral assemblies: (**a**,**b**) gold nanoparticles assembled by DNA origami in left- and right-handed nanohelices. (**a**) Schematics, (**b**) TEM image. Reproduced with permission from Kuzyk et al. [53] Copyright 2012 Springer Nature. (**c**–**f**) Detection of Amyloid Fibrils using gold nanorods. (**c**) TEM image and (**d**) cryo-TEM tomography reconstruction of a composite α-synuclein fiber showing the 3D chiral arrangement of Au nanorods; (**e**) extinction and (**f**) CD spectra of Au nanorods monitored 30 min after the addition of 30 μL of purified brain homogenates from healthy (black) and Parkinson-disease-affected (red) patients Adapted with permission from Kumar et al. [60] Copyright 2018 National Academy of Sciences.

One of the common approaches for arranging plasmonic nanoparticles in a chiral fashion is by using DNA molecules [51,52,53,54,55] (see Figure 3). Various types of DNA-based chiroplasmonic nanostructures have been covered in recent review [56]. Besides, promising results have been achieved using peptide-directed assembly of gold nanoparticles into single-helix [57,58] and double-helix superstructures [59].

Liquid crystals can as well be used to arrange metallic nanoparticles in chiral assemblies. The formation of silver helical nano-aggregates has been realized in cholesteric liquid crystalline phase based on cholesterol and its mesogenic derivatives incorporating silver nanoparticles of 2.5 nm in size [61,62,63]. Such structures have been obtained due to the specific interactions of ligand molecule’s functional groups with surface atoms of silver nanoparticles, followed by spiral self-organization of the hybrid system as a whole according to helical ordering of ligand molecules in cholesteric liquid crystalline mesophase. In case of thiocholesterol ligand the concentrated organosols of such aggregates possess high optical activity demonstrated by CD spectra. Optical spectra of these systems exhibit absorption near 430–450 nm, attributed to LSPR bands of silver nanoparticles, and a long-wavelength absorption at 700–1000 nm, characteristic to linear aggregates. This near-infrared region is suitable to perform in vivo imaging and biosensing.

Anisotropic plasmonic nanoparticles, such as nanorods, offer some additional advantages, including better focusing of the near field and high sensitivity to mutual orientation of the particles [50]. Generation of optical activity in a dimer of gold nanorods has been studied theoretically, showing that bonding and anti-bonding hybridized plasmonic modes contribute to CD signal [64]. Side-by-side self-assembly of gold nanorods, initiated by immobilized DNA oligomers in the PCR process, resulted in the formation of dimers and larger aggregates with strong chiroptical activity [65], which allowed achieving attomolar detection of DNA [66]. The chiral response originated from the small twist between nanorods. During the self-assembly process, both enantiomers have been formed, but one of them was thermodynamically more favorable. Glutathione molecules facilitated by cetrilammonium bromide micelles directed the self-assembly of gold nanorods into formation of chiral nanochains with end-to-end contacts [67].

It has been demonstrated that strong chiroptical signal can be generated by plasmonic nanorods assembled in three-dimensional helical structure by supramolecular fibers, such as anthraquinone-based oxalamid [68]. DNA origami approach offers unprecedented control over the plasmonic superstructure, enabling the dynamical switching between different nanorod helix configurations, leading to the change in chiroptical response [69].

Other biomolecular templates can be also used to arrange plasmonic nanoparticles in 3D chiral assemblies, such as proteins or their aggregates. Thus, the detection of α-synuclein amyloid fibrils by the CD from the helically arranged gold nanorods has been reported [60]. At the same time, no chiroptical activity has been detected when only monomers of α-synuclein was present. The technique can be further extended for the detection of infectious recombinant prions.

In some cases, aggregation of plasmonic nanoparticles, leading to drastic optical changes, can be initiated by only one of the enantiomers. For example, it has been reported that D-glutamic acid leads to aggregation of CTAB-capped gold nanorods leading to a significant color change, whereas there almost no change for L-enantiomer [70]. Similarly, enantioselective detection of D-cysteine by silver nanoparticles has been demonstrated recently in solution and on special bacterial cellulose matrix [71].

## 4. Enhanced Near-Field Chirality

To amplify the CD signal of a chiral molecule, the differential absorption of chiral molecule must be increased. Analytically, the light absorption rate can be estimated using the following relation [72]:

(1) A∝ω2α″|E˜|2+χ″|B˜|2−G″ωImE˜*·B˜

where ω is the angular frequency of electromagnetic wave, $\alpha^{\prime \prime },\chi^{\prime \prime }$ and $G^{\prime \prime }$ are the imaginary parts of electric polarizability, magnetic susceptibility and mixed electric-magnetic polarizability of the molecule, and $\tilde{E}$ and $\tilde{B}$ are the complex electric and magnetic field, respectively. One can see that CD is defined by the second term, which is determined by both the intrinsic chirality of a molecule ($G^{\prime \prime }\neq 0$) and by local electromagnetic field (ImE˜*·B˜), which needs to have non-orthogonal components of electric and magnetic field vectors.

Therefore, to enhance the CD response of a chiral molecule one can modify the electromagnetic field to maximize the local optical chirality *C*, the quantity originally introduced in 1964 [73] and recently revisited [72].

(2) C=−ϵ0ω2ImE˜*·B˜

The following subsections review the research directed at constructing plasmonic systems with enhanced near-field chirality and various examples of biosensing using these nanostructures.

### 4.1. Biosensing with 3D Chiral Nanostructures

**Figure 4:**
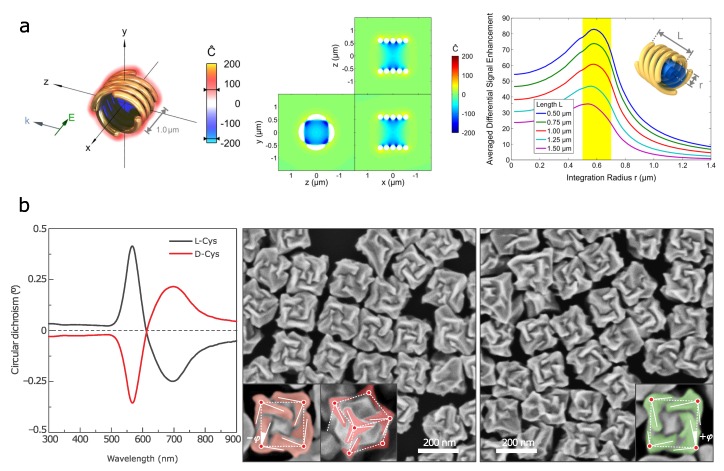
3D chiral plasmonic nanostructures: (**a**) simulated four-helix gold nanostructure showing strongly confined optical chirality within the helices (left, middle) and the graph presenting the enhancement of differential absorption signal depending on the radius of integration, for different length of a structure (right). Adapted with permission from Schaferling et al. [76] Copyright 2014 American Chemical Society. (**b**) Three-dimensional plasmonic helicoids controlled by cysteine chirality transfer: CD spectra and SEM images of chiral nanoparticles synthesized using L-Cys and D-Cys. The insets highlight the tilted edges (solid lines), cubic outline (dashed lines) and tilt angles (−ϕ and +ϕ). Reproduced with permission from Lee et al. [77] Copyright 2018 Springer Nature.

Much efforts have been made to increase local optical chirality *C* (Equation (2)) by using plasmonic nanostructures with chiral shape. An archetype chiral shape—nanohelix—has been studied by several research groups both theoretically and experimentally. Numerical simulations have shown that arrays of gold nanohelices exhibit strong CD [74]. However, the chirality enhancement factor calculated for 400 nm high gold nanohelices with diameter of 400 nm and nanowire thickness of 80 nm was of the order of ten only, thus suggesting limited application of such structures in biosensing [75]. Later, it has been shown that the optical chirality enhancement factors can be increased up to two orders of magnitude in superstructures consisting of multiple helices [76]. The near field with enhanced chirality is confined within the helix, as demonstrated in Figure 4a.

Experimental fabrication of such nanohelices requires the use of rather sophisticated techniques, such as direct laser writing [78] or focused ion/electron beam deposition [79,80,81]. The latter approach allowed fine-tuning of the CD signal from IR to the visible range of the spectrum, as well as fabricating core-shell nanohelices with independently tunable dissymmetry factor *g* and the spectral position of CD [82].

Another way of using 3D chiral plasmonic nanohelices for sensing has been demonstrated recently [83]. Instead of exploiting the enhanced chirality of the near field, the CD signal of Pd nanohelices has been used to detect hydrogen content of 0.1–1%. Large-area arrays of Pd and hybrid Pd-Au nanostructures have been produced using nanoglancing angle deposition technique [84], which combines block copolymer micelle nanolithography [85] with glancing-angle deposition (GLAD) [86].

Chiral lattice and shape can be induced during synthesis by the adsorption of chiral organic molecules on growing nanocrystals. The formation of chiral Te and Se nanocrystals that can be used as a template for growing gold and silver telluride nanostructures has been demonstrated [87]. Recently, the growth of chiral gold nanoparticles of opposite handedness, induced by amino acids and peptides, has been presented [77]. The development of chiral morphology as a result of the different growth rates of the two oppositely chiral high-index planes of gold in the presence of L-Cys or D-Cys has been shown. Very different morphology of the nanocrystals was observed in the presence of L-glutathione (GSH). The structure and the CD spectra of these nanocrystal are presented in Figure 4b. The proposed mechanism involves specific adsorption of Cys or GSH on the high-index planes of growing particles.

### 4.2. Planar Chiral Nanostructures and Metasurfaces

**Figure 5:**
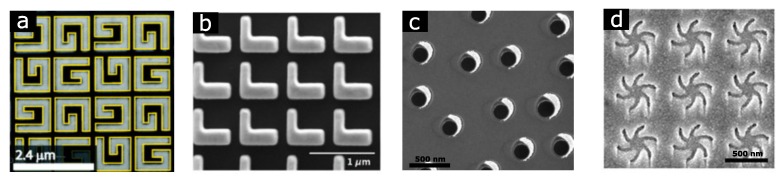
Examples of planar chiral nanostructures: (**a**) arrays of G-shaped gold nanoparticles. Adapted with permission from Valev et al. [89] Copyright 2009 American Chemical Society. (**b**) Arrays of L-shaped gold nanoparticles. Adapted with permission from Ye et al. [91] Copyright 2017 American Physical Society. (**c**) Short-ordered arrays of comma-shaped gold nanoparticles [94]. (**d**) Gold metafilm with arrays of “Shuriken” nanostructures. Adapted with permission from Kelly et al. [98] Copyright 2018 American Chemical Society.

Planar plasmonic nanostructures with the thickness much less than the wavelength of incident light are very attractive from the technological point of view, due to the potential of mass production using conventional lithographic techniques. Just as for the truly chiral shapes with enantiomers, which cannot be superimposed by any rotation in 3D space, the left- and right-handed enantiomers of a planar chiral shape are not superimposable by in-plane rotation. The handedness in the latter case is defined by the viewing side, resulting in the inverted CD spectra when planar structure is illuminated from the opposite normal direction. Unlike 3D helical structures, the differential absorption of LCP and RCP light by planar plasmonic nanostructures is originated from the difference in LSPR-induced near-field distributions [88]. Several 2D chiral plasmonic nanostructures have been studied (see Figure 5), such as G- [89], S- [90], L-shaped [91] nanostructures, gammadions [92,93], nanospirals [75], asymmetric nanocrescents [94] and staggered nanorods [95]. Similar effects have been reported for planar metamaterials comprising thin metal films with 2D chiral openings, such as nanoslit dimers [96,97].

Planar chiral plasmonic nanostructures generate chiroptical response, which can be highly sensitive to the presence and tertiary structure of biomolecules, as in the case of arrays of gold gammadion nanostructuress [99]. The difference in plasmonic peak shifts observed for RCP and LCP light upon adsorption of proteins $\Delta \Delta \lambda =\Delta \lambda_{RCP}-\Delta \lambda_{LCP}$ has been used as an analytic signal. Thus, for achiral adsorbed molecules this parameter becomes zero: ΔΔλ=0. The detection of the picogram levels of proteins has been reported using these structures. Moreover, different response was observed for proteins with different content of β-sheets, thus providing information about the structure of the adsorbed biomolecules. This feature has been studied in detail using chiral “shuriken”-like gold-covered indentations created on polycarbonate templates using injection molding method and gold deposition [100]. Two well-studied proteins, 5-enolpyruvylshikimate 3-phosphate synthase and shikimate kinase, capable for ligand-induced conformational changes, have been used as model systems. The far-field chiroptical properties were characterized by collecting the ORD spectra in reflection mode for linearly polarized incident light. The observed peak shifts demonstrated picogram level detection limit of the proteins. Conformational changes due to the protein binding with ligands could be followed by changes of the asymmetry factor ΔΔλ, demonstrating the sensitivity to tertiary and domain (quaternary) structure of proteins [101].

Later studies have demonstrated that these chiral plasmonic substrates enable discrimination of proteins which have similar structures but have primary sequences that differ by a single amino acid [102] and can provide information about structural order in complex biointerfaces [98].

Thus, the “superchiral” fields, generated by 2D-chiral nanostructures, provide the unique capability of probing the chirality of the adsorbed biomolecules at different levels, including molecular structure, conformation, orientation, and supramolecular ordering.

## 5. Outlook

In conclusion, the unique properties of plasmonic nanostructures open new exciting possibilities for enhanced sensing of molecular chirality. Among several experimental and theoretical studies in this area, several general approaches exist, each providing different advantages. Despite moderate CD enhancement factors, the formation of complexes with small achiral nanoparticles has proven to be useful for detection of peptides and small chiral organic molecules. An alternative approach based on formation/destruction of chiral plasmonic dimers, constructed with the use of target-specific aptamers, offers the possibility to detect a large range of biomolecules, including enzymes and proteins, in complex biological environments and even inside living cells. The formation of self-organized 3D chiral assemblies of achiral plasmonic nanoparticles can be used to efficiently detect the supramolecular biological structures, such as amyloid fibrils, for early diagnostics of various related diseases. Complicated fabrication and moderate optical chirality enhancement hinder the application of 3D chiral plasmonic nanostructures, whereas planar chiral metasurfaces, which can be conveniently produced using lithographic methods, demonstrate high potential for ex vivo detection of chiral biomolecules, additionally allowing to probe their tertiary and domain structure.

The field of chiral plasmonic biosensing is still at its development stage, and one can expect more exciting results to be published in the near future. A clearer understanding of the nature of interactions of chiral molecules with plasmon-induced near fields, developed in the previous years, will facilitate the search for more advanced nanosystems, including nanostructures with new geometries and their complex assemblies; the use of new, first of all, dielectric [103], materials, as well as hybrid materials and alloys with tailored dielectric functions [104] for enhanced sensitivity of chiral biosensors; using combinations of different types of chiral nanostructures for simultaneous detection of chirality at different scale or for the detection of different chiral molecules in complex biological media.

## Abbreviations

The following abbreviations are used in this manuscript:

AFP	alpha-fetaprotein
CD	Circular Dichroism
CPL	Circularly Polarized Light
EBL	Electron Beam Lithography
GLAD	Glancing-Angle Deposition
LCP	Left Circular Polarization
LSPR	Localized Surface Plasmon Resonance
ORD	Optical Rotatory Dispersion
PEF	Plasmon-Enhanced Fluorescence
PSA	Prostate-specific antigen
RCP	Right Circular Polarization
SEIRA	Surface-Enhanced Infrared Absorption
SERS	Surface-Enhanced Raman Scattering
SPR	Surface Plasmon Resonance
